# Tumor-shrinking effects of enfortumab vedotin between primary urothelial carcinoma and metastatic organs

**DOI:** 10.3389/fonc.2024.1493922

**Published:** 2025-01-29

**Authors:** Daiki Ikarashi, Tatsuya Kawamura, Keita Ogasawara, Yumeka Arakawa, Arisa Machida, Ayato Ito, Ei Shiomi, Shigekatsu Maekawa, Renpei Kato, Mitsugu Kanehira, Jun Sugimura, Wataru Obara

**Affiliations:** Department of Urology, Iwate Medical University School of Medicine, Iwate, Japan

**Keywords:** durability, enfortumab vedotin, primary, metastatic organ, tumor shrinkage

## Abstract

**Objective:**

This study aimed to determine and compare the tumor shrinkage rate and its durability by enfortumab vedotin treatment between primary urothelial carcinoma and metastatic organs.

**Methods:**

We retrospectively evaluated the tumor shrinkage rate in 39 Japanese patients treated with enfortumab vedotin for advanced urothelial carcinoma. We also evaluated the periods of tumor shrinkage maintenance (the period when best response was maintained) and regrowth (the period from best response to tumor growth confirmation) between primary and metastatic organs.

**Results:**

Measurable metastatic organs included the lung in 17, lymph node in 22, liver in 6, and bone in 5 cases. Primary lesion was detected in 20 cases. The mean tumor shrinkage rates for lung, lymph node, liver, and bone metastases and primary sites were 21% (−212 to 100), 13% (−130 to 86), −8.5% (−158 to 85), −64% (−250 to 21), and 22% (−38 to 79), respectively. The tumor shrinkage was maintained for 5.9 (0.7–14) months in lung metastases, 8.3 (2.6–14.5) months in lymph node metastases, 3.6 months in liver metastases, 0.7 months in bone metastases, and 1.8 (0.7–5.4) months in primary sites, and the period of regrowth was 7.3 (2.2–19.4), 4.8 (2.0–8.9), 2.8, 6.5, and 2.5 (1.1–5.9) months, respectively.

**Conclusions:**

Enfortumab vedotin showed significant tumor shrinkage in the primary tumor, lung metastases, and lymph node metastases, whereas the durability of tumor shrinkage was limited in the primary tumor.

## Introduction

Urothelial carcinoma (UC) is one of the most common cancers arising from the renal pelvis, ureter, bladder, or proximal urethra, and bladder cancer accounts for approximately 95% of the cases (1). Approximately 20% of invasive UC cases are metastatic or unresectable, and sequential therapy with chemotherapy and immune checkpoint inhibitors (ICIs) has been used to improve their prognosis (2–5).

The therapeutic strategy of metastatic UC has expanded in recent years. Most recently, enfortumab vedotin (EV), an antibody–drug conjugate (ADC), has been used for treating unresectable or metastatic UC after chemotherapy and ICI treatment, resulting in remarkable advances for previously treated UC in clinical practice and improvement in patient’s prognosis regardless of being a third-line therapy or later. In an EV-301 trial basis for FDA approval, EV demonstrated an objective response rate (ORR) of 40.6% and a disease control rate (DCR) of 71.9%; patients taking EV also showed improvement in overall survival (OS) and progression-free survival (PFS) compared with control groups (6). Moreover, a Japanese subgroup analysis of EV-301 trials confirmed the efficacy of EV treatment (7).

However, while EV has been reported to be valid, its efficacy and durability are reportedly limited, with few data on the difference in efficacy, especially between primary and metastatic sites. Miyake et al. (8) examined the rate of organ-specific response to EV treatment and demonstrated no notable differences in ORR among primary and metastatic lesions, ranging from 40%–60% after 3 months of EV treatment. Moreover, the response was not durable in all evaluated target lesions, given that the ORR decreased over time. Among metastases, liver metastases were not observed in any case of response at 6 months after EV administration, indicating differences in response and durability. Based on these results, therapeutic strategies to enhance the durability of tumor response might be beneficial to patients treated with EV. Therefore, our study aimed to identify the differences in antitumor efficacy and durability of EV treatment between primary and metastatic sites.

## Materials & methods

### Patient data

We retrospectively evaluated 39 patients with unresectable or metastatic UC treated with EV after chemotherapy and ICI treatment at the Iwate Medical University Hospital between January 2017 and March 2024. Several clinical factors, including age, sex, performance status, pathological status, and post-medical history, were recorded. This study obtained approval from the Iwate Medical University Institutional Review Board (approval number: MH2023-059) and conformed to the principles of the Declaration of Helsinki.

### Treatment and response

EV was initially administered at 1.25 mg/kg intravenously on days 1, 8, and 15 of a 28-day cycle. This treatment strategy is the established dosing method for EV covered by the Japan health insurance system. Generally, all patients underwent computed tomography (CT) every 8 weeks according to the EV-301 trial, and tumor response was evaluated according to the Response Evaluation Criteria in Solid Tumors version 1.1 (9). The response categories were complete response (CR), partial response (PR), stable disease (SD), and progressive disease (PD). We also considered PD for patients experiencing clinical progression or cancer-specific death, as determined by the attending physician during the last follow-up. ORR was defined as CR + PR rate, and DCR was defined as ORR + SD rate.

### Tumor diameter measurement and duration of response

In tumor diameter measurement, we measured tumor lesion sizes on the CT scan of primary and each metastatic organ. One researcher (D.I.) and two radiologists read independently measured tumor lesion size for each CT scan. Regarding measurement of bone metastasis, our study included only the patients with osteolytic lesions who had a measurable soft tissue component. Tumor shrinkage rate was defined as the percentage change from baseline (before EV treatment) in the sum of the diameters of the measurable target lesion at the best response among primary and each metastatic organ, which is shown in waterfall plots. Negative percentages were used when the tumor was enlarged.

The durability of EV was evaluated for the periods indicated as the period of shrinkage maintenance and the period of tumor re-growth. The period of shrinkage maintenance was the period during which EV was administered, and the best response had been maintained. The period of tumor re-growth was the period from best response to tumor growth confirmation in CR, PR and SD cases before a determination of PD (**Supplementary Figure 1**). A measurable increase on CT from the sum of the maximum tumor diameter at best response in primary and each metastatic organ indicated tumor regrowth.

### Statistical analysis

Using the Kaplan–Meier method, we calculated PFS and OS from the date of the initial EV dose. Furthermore, the tumor shrinkage rate was calculated and compared between the primary organ and each metastatic organ using the analysis of variance and Tukey’s honestly significant difference test.

All statistical data were analyzed using JMP software (SAS Institute Inc., Cary, NC, USA). For all statistical comparisons, differences with a *p*-value less than 0.05 were considered statistically significant.

## Results

**Figure 1:**
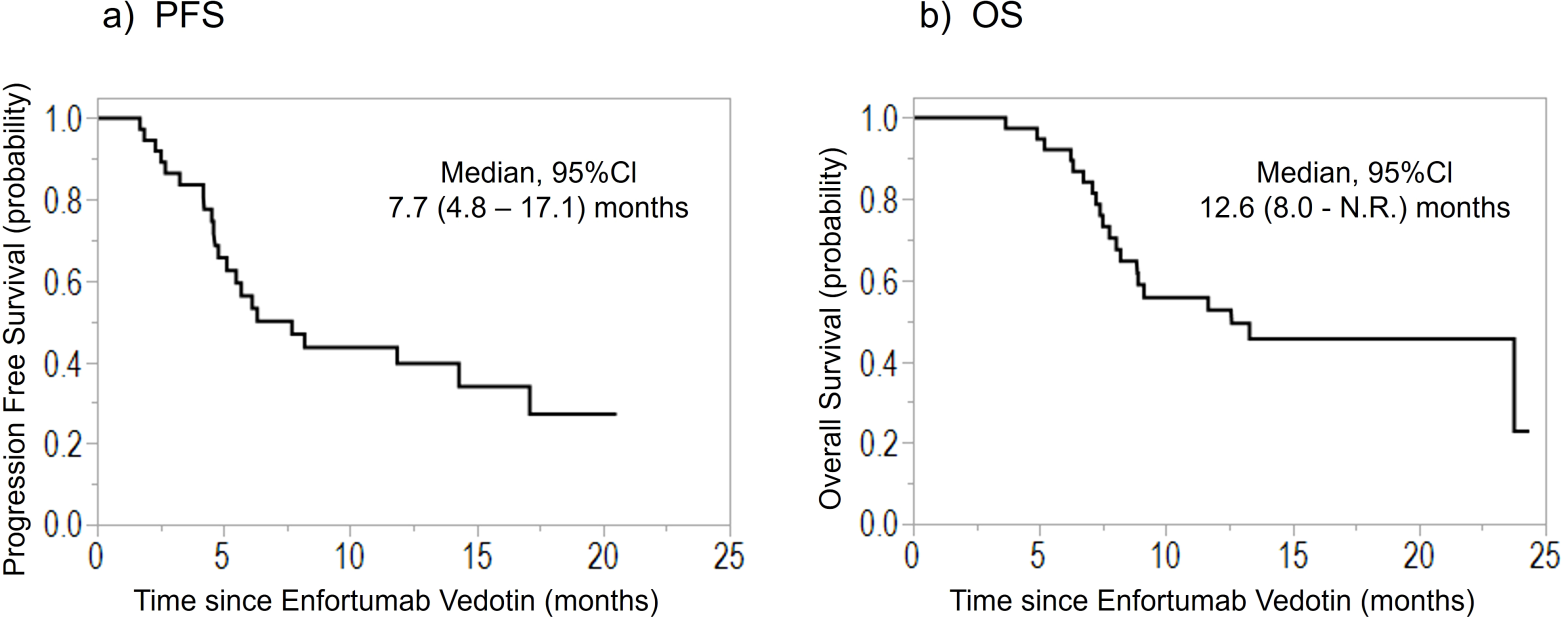
Kaplan–Meier curves of the **(A)** PFS and **(B)** OS of patients treated with enfortumab vedotin for previously treated advanced urothelial carcinoma. PFS, progression-free survival; OS, overall survival; CI, confidence interval; N.R., not reached.

**Table 1** lists patients’ characteristics. The median age was 69 (44–83) years. Among the 39 included patients, 17 (44%) underwent radical surgery before EV introduction. When avelumab was included in the treatment line, EV was introduced as the third-line therapy in most cases (85%). After EV treatment initiation, the median follow-up duration was 8.9 (3.7–24.4) months, and the median PFS and OS were 7.7 months (95% confidence interval [CI], 4.8–17.1) and 12.6 months (95% CI, 8.0–unavailable), respectively (**Figure 1**). Moreover, the ORR was 44%, and DCR was 85%. The best response was CR in 2 (5%), PR in 15 (39%), SD in 16 (41%), and PD in 6 (15%) patients.

**Table 1:** Patients characteristics.

Valuable	Level	All patients
		(n=39)
Age, years	median	69 (41-82)
Sex, n (%)	Male	32 (82%)
	Female	7 (18%)
Performance Status	0	25 (64%)
	1	8 (21%)
	2	6 (15%)
Primary lesion	Bladder	21 (54%)
	UTUC	18 (46%)
Histology	Pure UC	35 (90%)
	Variant histology	4 (10%)
Tumor grade, n (%)	Low grade	4 (10%)
	High grade	29 (74%)
	Unknown	6 (15%)
Metastatic lesion	Lung	19 (49%)
	Lymph node	23 (59%)
	Liver	6 (15%)
	bone	8 (21%)
Radical surgery	Yes	17 (44%)
	No	22 (56%)
Treatment line	3^rd^ line	33 (85%)
	≧4^th^ line	6 (15%)
Prior chemotherapy regimen	GC	28 (72%)
	GCa	11 (28%)
Prior ICI regimen	Pembrolizumab	22 (56%)
	Avelumab	12 (31%)
	Nivolumab	4 (10%)
	Atezolizumab	1 (3%)

UC, urothelial carcinoma; UTUC, upper tract urothelial carcinoma; ICI, immune checkpoint inhibitor; GC, gemcitabine + cisplatin; GCa, gemcitabine + carboplatin.

**Figure 2:**
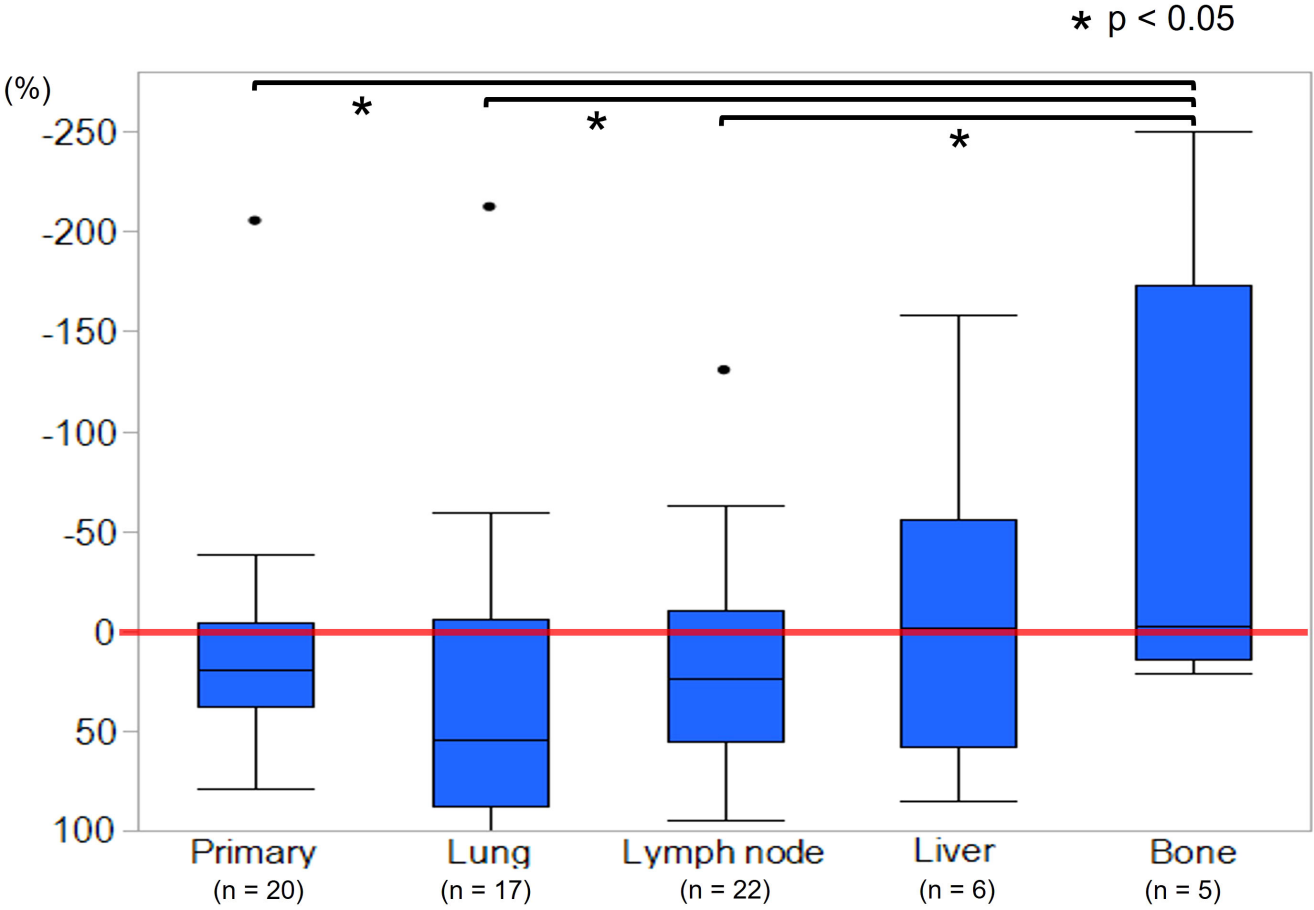
Mean tumor shrinkage rate at best response between primary tumor, lung, lymph node, liver, and bone metastases. The tumor shrinkage rates of the primary site, lung metastases, and lymph node metastases were significantly higher than that of bone metastases (*p* < 0.05).

**Figure 3:**
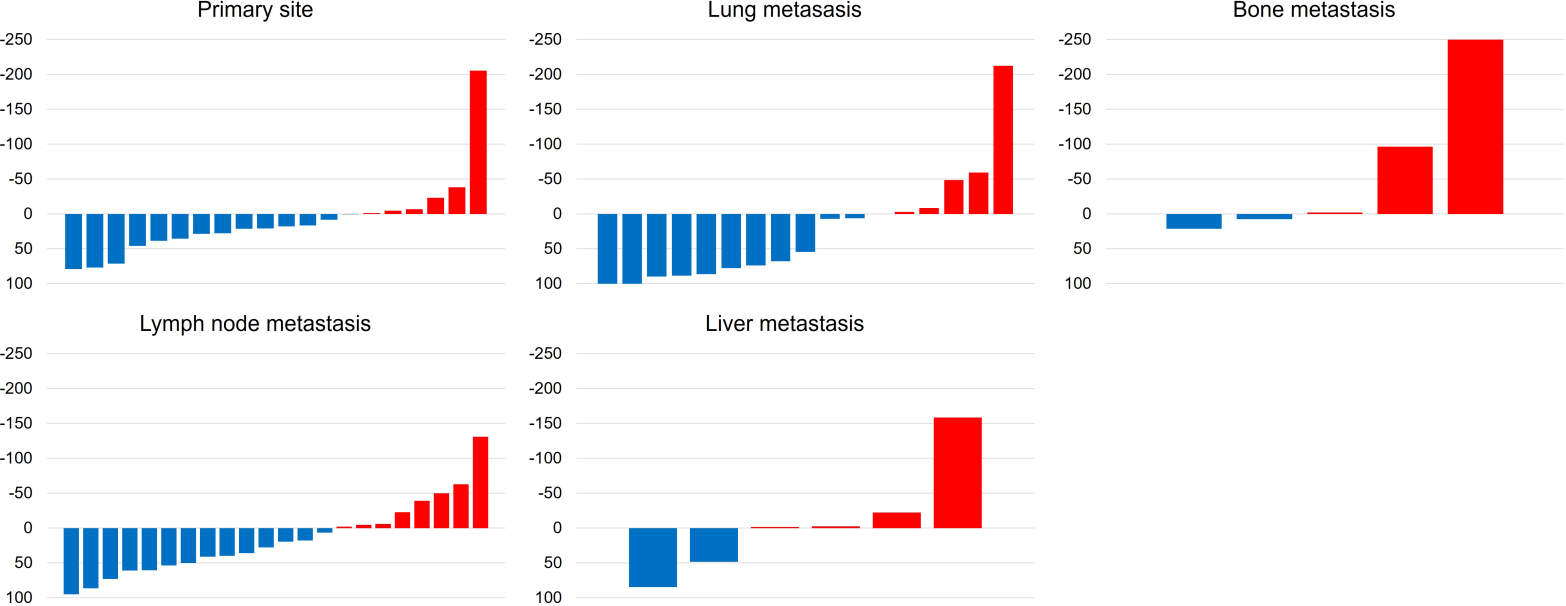
Waterfall plots demonstrate the change from baseline to the best response (sum of target legion diameters) by enfortumab vedotin between primary tumor, lung, lymph node, liver, and bone metastases according to the Response Evaluation Criteria in Solid tumor (RESIST) version 1.1.

Measurable metastatic organs were the lung, lymph node, liver, and bone in 17, 22, 6, and 5 cases, respectively, including duplicate cases. Primary lesion was confirmed in 20 cases. **Figure 2** shows the mean tumor shrinkage rates at best response, with 22% (−38 to 79) for the primary tumor, 21% (−212 to 100) for lung metastases, 13% (−130 to 86) for lymph node metastases, −8.5% (−158 to 85) for liver metastases, and −64% (−250 to 21) for bone metastases. The tumor shrinkage rate did not significantly differ between the primary tumor and each metastatic organ (*p* = 0.1553), but significant differences were noted between the primary tumor and bone metastasis (*p* = 0.0373), between lung metastasis and bone metastasis (*p* = 0.0153), and between lymph node metastasis and bone metastasis (*p* = 0.0244). The waterfall plot showed some degree of tumor shrinkage by EV treatment in 14 (70%) primary tumor cases as well as in 12 (70.6%), 14 (63.6%), 2 (33.3%), and 2 (40%) cases of lung, lymph node, liver, and bone metastases, respectively (**Figure 3**).

Moreover, shrinkage was maintained longest at 8.3 (2.6–14.5) months for lymph node metastases, followed by 5.9 (0.7–14) months for lung metastases, 3.6 months for liver metastasis, 1.8 (0.7–5.4) months for the primary lesion, and 0.7 months for bone metastases. As for the period of regrowth, it was shortest at 2.5 (1.1–5.9) months for the primary lesion, followed by 2.8, 4.8 (2.0–8.9), 6.5, and 7.3 (2.2–19.4) months for liver, lymph node, bone, and lung metastases, respectively (**Table 2**).

**Table 2:** The period of tumor shrinkage maintenance and tumor re-growth.

Lesion	Total	Shrinkage case	Maintained shrinkage	Period of maintenance	Re-growth	Period of re-growth
	(n*)	(n)	(n)	(month)	(n)	(month)
Primary	20	14 (70%)	5	1.8	9	2.5
				(0.7-5.4)		(1.1-5.9)
Lung	17	12 (71%)	6	5.9	6	7.3
				(0.7-14)		(2.2-19.4)
Lymph node	22	14 (63%)	6	8.3	8	3.6
				(2.6-14.5)		(1.1-6.5)
Liver	6	2 (33%)	1	3.6	1	2.8
Bone	5	2 (40%)	1	0.7	1	6.5

*Measurable each organ including duplicate cases

The tumor shrinkage with EV was observed highest in lung metastasis, followed by primary and lymph node metastasis. The period of regrowth was shortest in primary lesion compared with metastatic organs.

The average total tumor burden among primary and each metastatic organs according to each treatment lines were described in **Table 3**. The average tumor burden before EV was highest in the primary sites (43.7mm), followed by the lung metastasis (39.2mm). With regard to the primary tumor, the tumor load increased for each treatment line.

**Table 3:** Baseline average total tumor size according to each treatment lines.

Lesion	Average total tumor size (mm)		
	1st line	2nd line	before EV
Primary	30.3	34.7	43.7
Lung	33.3	42.5	39.2
Lymph node	20.5	25.6	17.5
Liver	36	31	28.3
Bone	23.3	21.6	20.3

EV, enfortumab vedotin.

The average total tumor burden (mm) among primary and each metastatic organs according to each treatment lines.

## Discussion

This study, which was conducted in real-world clinical practice, have significantly higher PFS (7.7 months), ORR (44%), and DCR (85%) than the EV-301 trial, but the OS (12.6 months) is similar between these two studies. Recently, several real-world clinical data on EV in Japan have been reported. Fukuokaya et al. (10) showed that the PFS, OS, ORR, and DCR were 6.0 months, 14.5 months, 50.5%, and 73.8%, respectively, in 103 EV-treated patients; this sample size is currently the largest Japanese cohort ever included in a multicenter study. Compared with other Japanese cohorts of retrospective reports of EV, our results show comparable outcomes for EV treatment (8, 11, 12). In addition, one prospective study in Japan reported that the PFS and OS were 6.9 and 13.5 months, and the ORR and DCR were 52.9% and 73.5%, respectively (13).

The present study showed a certain degree of tumor shrinkage in both primary and metastatic organs by EV treatment. Especially, primary lesion, lung metastases, and lymph node metastases had significantly higher tumor shrinkage rates than bone metastases. Although there were outliers in the primary tumor and lung and lymph node metastases in the water fall plot for tumor shrinkage rate, these were PDs in the same patient. This result is clinically important because the addition of local treatment becomes significant when the EV effect differs for each target organ. EV consists of a monoclonal antibody targeted against Nectin-4 that is conjugated to monomethyl auristatin E (MMAE) (14). Nectin-4 is highly expressed in UC and is a potential biomarker for EV treatment (15, 16). Furthermore, Nectin-4 expression intensity and changes in expression with previous treatment reportedly correlate with EV efficacy (17). In other ADCs, the expression level of target proteins, such as HER2, is also associated with ADCs’ therapeutic effect (18). The difference in tumor shrinkage rate between primary and metastatic organs may be explained by the differences in Nectin-4 expression in each organ, and Nectin-4 expression would be different between primary and metastatic organs during EV administration. Nectin-4 expression reportedly demonstrates no correlation between primary tumors and matched lymph node metastases, and chemotherapy significantly downregulates the respective Nectin-4 expression (17, 19). Therefore, Nectin-4 expression changed during EV administration because of prior chemotherapy and ICI treatment, and no correlation in this expression would exist between primary and matched metastatic organs. Additionally, we hypothesize these mixed reactions may be due to the heterogeneous tumor clonality between the primary and metastatic sites. In the latest report, whole exome and RNA sequencing for biopsies of several metastases’ sites were performed and compared the primary UC (20). They demonstrated that paired samples analysis reveals subtype heterogeneity and temporal evolution such as FGFR3 alterations. Our study also showed baseline total tumor volume among each organ according to treatment lines. The primary tumor increased with each treatment line, with the highest tumor volume before EV administration compared with metastatic organs. This result suggests pre-treatment tumor volume may be related to EV resistance. The tumor volume related to bladder field cancerization which leads worse prognosis. Bladder field cancerization was associated with cancer angiogenesis, proliferation, and drug resistance (21). In real world clinical practice, EV monotherapy was difficult to select based on the site of metastasis because EV monotherapy is a late-line treatment. However, personalized medicine based on the genetic mutation of the disease lesion may provide a new benchmark for use in EV monotherapy.

Our study demonstrated not only tumor shrinkage rate but also its durability between primary and each metastatic organ, highlighting clinical importance. In primary tumor, lung metastases, and lymph node metastases, where tumor shrinkage rate was high, a sustained long-term response was noted in lung and lymph node metastases, and a short-term response in primary tumors. Thus, the emergence of resistance for EV may differ between primary and metastatic organs. These findings indicate that EV monotherapy is a good candidate for tumor shrinkage in clinically advanced patients despite Late line, and that strategies for EV resistance are clinically important. ADCs’ resistance mechanisms, including antigen-related resistance, internalization failure, and impaired lysosomal function, were reviewed (22). Drug exclusivity, one of the resistance mechanisms, is a factor contributing to the difference in the persistence of the EV effect between primary and metastatic organs. The overexpression of ATP-binding cassette transporters associated with drug extrusion from cells is essential in chemotherapy resistance, and in many cancer types, transporter expression has been associated with less response to chemotherapy (23). Moreover, the expression of transporter differs between primary and metastatic organs (24). Therefore, long-term disease control may be achieved by developing therapeutic strategies that can overcome these resistance mechanisms. We previously reported two cases of successful durable response to radiotherapy to primary lesions with EV under controlled lung or lymph node metastases for metastatic UC, suggesting that additional radiotherapy to EV is an effective treatment option for patients with metastatic UC with controlled disease (25). The antitubulin agent MMAE, the key drug of EV, has a potential for radiosensitization. MMAE radiosensitization has shown to be both schedule- and dose-dependent, directly correlating with the cell accumulation in the G2/M checkpoint (26). Similar to MMAE, antitubulin chemotherapeutic agents, such as paclitaxel, induce a strong arrest of cells in the G2/M cell cycle phase as radiosensitizers and have been used along with radiotherapy in real-world clinical practice (27). Furthermore, ADCs with peptides conjugated to radiosensitizing MMAE products tumor specific CD8^+^ T cells dependent durable tumor control of irradiate tumors and immunologic memory; consequently, these agents facilitate the tumor immune infiltrate to potentiate ICIs in combination radiotherapy (28). Abscopal effect might also be expected because the condition of EV treatment is also post-treatment with ICIs accordingly (29). Although these results are hypothetical and need further validation, we believe that these results demonstrate the potential efficacy of EV in combination therapy with irradiation and ICIs, and are expected to broaden treatment strategies.

Our study has several limitations. First, our study has a small sample size and a retrospective study design. Therefore, a prospective study with a large cohort should be conducted in the future. Second, this study has relatively fewer cases of liver and bone metastases than the other metastatic organs, and even fewer cases of tumor shrinkage. Minato, et al. reported organ specific tumor response to EV for metastatic UC, and the tumor shrinkage rate was lower for bone metastases than the primary and other metastatic organs similar to our results (30). A sub analysis of the EV-301 trial showed that liver metastasis benefits from EV; hence, more cases need to be accumulated. Furthermore, the type of prior chemotherapy and ICIs used prior to EV and other factors may have a significant impact on tumor response and durability of each organs. Therefore, we have added a comparison between the two groups according to the type of prior chemotherapy and ICIs: the chemo-pembrolizumab group; which failed first-line therapy, and chemo-other ICIs group; which partially responded to first-line therapy or adjuvant use. The results showed no difference between the two groups (**Supplementary Table 1**). Third, Nectin-4 expression was not evaluated and compared between primary tumor and metastatic organs. In actual practice, collecting tissues from primary and metastatic sites is difficult during EV administration. However, our results showed that the long-term disease control may be possible by combining local treatments in primary sites, and our data could contribute to developing new therapeutic strategies, such as presurgical setting or bladder sparing therapy, when EVs are used as a primary treatment (31).

In conclusion, EV treatment has been effective in real-world clinical practice. Although cases of significant response have been observed in both primary and metastatic diseases, the durability of this response is limited. Especially in primary sites, the period of re-growth in the primary lesion was shorter than other metastatic organs. These findings are useful in developing treatment strategies that may enhance the durability of tumor response. Long-term disease control may be possible by combining EV with local treatment under controlled metastatic organs.

## Data Availability

The original contributions presented in the study are included in the article/**Supplementary Material**. Further inquiries can be directed to the corresponding author.
