# Supplementary figures and images for: Tumor-shrinking effects of enfortumab vedotin between primary urothelial carcinoma and metastatic organs
Source: Front Oncol. 2025 Jan 29;14:1493922. doi: 10.3389/fonc.2024.1493922 (PMC11813790; doi:10.3389/fonc.2024.1493922)

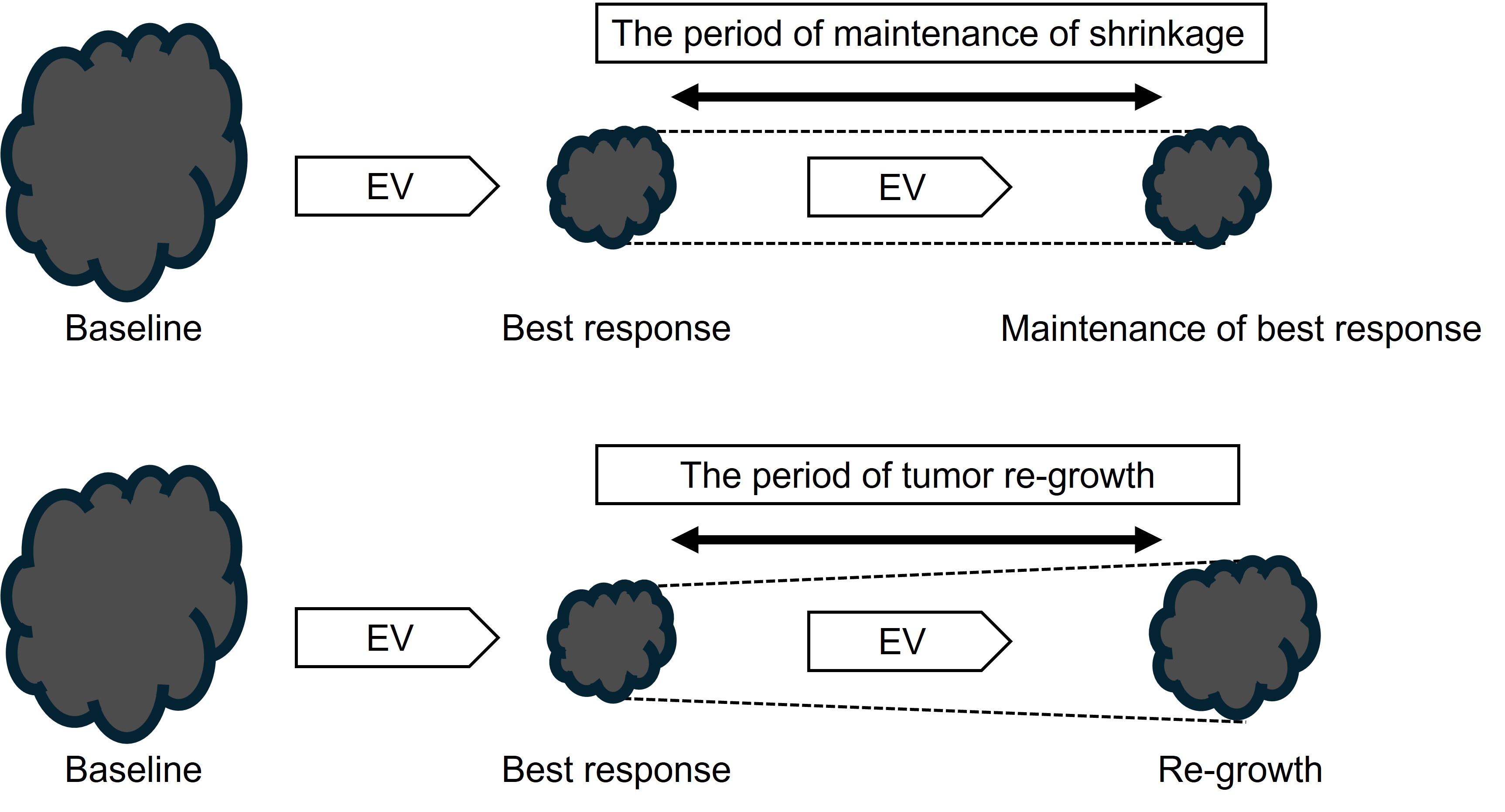

Supplement: Supplementary file 2 [file Image1.jpeg]
